# Understanding vegetation phenology responses to easily ignored climate factors in china's mid-high latitudes

**DOI:** 10.1038/s41598-024-59336-5

**Published:** 2024-04-16

**Authors:** Qianfeng Wang, Huixia Chen, Feng Xu, Virgílio A. Bento, Rongrong Zhang, Xiaoping Wu, Pengcheng Guo

**Affiliations:** 1 College of Environmental and Safety Engineering/The Academy of Digital China (Fujian), Fuzhou University, Fuzhou, 350116 China; 2 Faculdade de Ciências, Instituto Dom Luiz, Universidade de Lisboa, 1749-016 Lisboa, Portugal; 3 Key Lab of Spatial Data Mining & Information Sharing, Ministry of Education of China, Fuzhou, 350116 China; 4 School of Ecology and Environment, Hainan University, Haikou, 570228 China; 5 Hainan Guowei Eco Environmental Co., Ltd, Haikou, 570203 China

**Keywords:** Phenology, Vegetation, Preseason, Climate change, Climate-change ecology, Phenology

## Abstract

Previous studies have primarily focused on the influence of temperature and precipitation on phenology. It is unclear if the easily ignored climate factors with drivers of vegetation growth can effect on vegetation phenology. In this research, we conducted an analysis of the start (SOS) and end (EOS) of the growing seasons in the northern region of China above 30°N from 1982 to 2014, focusing on two-season vegetation phenology. We examined the response of vegetation phenology of different vegetation types to preseason climatic factors, including relative humidity (RH), shortwave radiation (SR), maximum temperature (Tmax), and minimum temperature (Tmin). Our findings reveal that the optimal preseason influencing vegetation phenology length fell within the range of 0–60 days in most areas. Specifically, SOS exhibited a significant negative correlation with Tmax and Tmin in 44.15% and 42.25% of the areas, respectively, while EOS displayed a significant negative correlation with SR in 49.03% of the areas. Additionally, we identified that RH emerged as the dominant climatic factor influencing the phenology of savanna (SA), whereas temperature strongly controlled the SOS of deciduous needleleaf forest (DNF) and deciduous broadleaf forest (DBF). Meanwhile, the EOS of DNF was primarily influenced by Tmax. In conclusion, this study provides valuable insights into how various vegetation types adapt to climate change, offering a scientific basis for implementing effective vegetation adaptation measures.

## Introduction

Vegetation phenology, denoting the recurrent seasonal fluctuations in vegetation growth, constitutes a crucial indicator of vegetation development with profound implications for terrestrial carbon balance and ecosystem productivity^1–5^. As an exceptionally responsive bio-indicator, vegetation phenology is a particularly important asset to climate change, making it an invaluable tool for monitoring ecological responses to shifting climate conditions^3,6–8^. Over recent decades, the dynamics of vegetation phenology have garnered global attention, highlighting the intricate interplay between phenology and climatic factors^2,9,10^. Consequently, comprehensive investigations into ecosystem responses to climate variables are essential for deepening our comprehension of vegetation dynamics within the context of climate change^11^.

The influence of climate change on phenology has emerged as a focal point in vegetation phenology research. Uncertainty about future climate change is growing^12–15^. Previous studies have explored the effect of temperature and precipitation on vegetation phenology^4,16,17^. However, it is crucial to acknowledge that external climatic drivers affecting vegetation phenology encompass not only temperature and precipitation but also factors such as relative humidity, solar radiation, maximum and minimum temperatures. These variables engage in complex interactions that either facilitate or constrain natural vegetation growth^13,17–21^. While temperature's primary role in influencing vegetation phenology is widely recognized, the distinct impacts of relative humidity and solar radiation remain less clear^3,22–24^. Notably, relative humidity significantly influences nutrient phenology in specific vegetation types^25,26^, while solar radiation, specifically shortwave radiation, exerts considerable influence by modulating photosynthetically active radiation. Some studies suggest that increased photosynthetically active radiation advances leaf emergence and retards leaf senescence^27,28^. Additionally, while temperature is acknowledged as the primary driver of vegetation phenology, many studies have proposed asymmetric effects of maximum and minimum temperatures on phenological patterns^29–31^. Furthermore, various vegetation types exhibit diverse responses to climatic elements, contributing to spatial heterogeneity both between and within regions^32–35^. In sum, examining the influence of these neglected climatic factors on phenology across different vegetation types is imperative.

Remote sensing identification of vegetation phenology is mostly based on seasonal changes in vegetation indices. Despite the emergence of various vegetation indices, the Normalized Differences Vegetation Index (NDVI) remains a cornerstone in vegetation phenology research due to its global acceptance, simplicity, and efficacy^36,37^. In addition, the initial remotely sensed vegetation indices need smoothing and noise reduction due to their inherent variability^38^. Among the various smoothing methods, the empirical method is the simplest to apply, but it is usually sensitive to empirical parameters such as noise threshold and synthesis cycle length^39^. Although the data conversion method is more flexible in capturing high-frequency changes in the curves, it has poor smoothing ability and relies too much on ground-based phenology data^40,41^. The curve-fitting method, on the other hand, uses a mathematical function to estimate the time-series trajectory of vegetation growth without the need to set empirical thresholds in advance, which is relatively more objective^42^. Among them, the Savizky-Golay filter can maximize the retention of data information while eliminating noise interference, which makes it particularly well-suited for extracting climate-related insights from different vegetation^43,44^. Commonly used methods for determining key phenological parameters include the threshold method and the method for detecting changes in vegetation indices. The threshold method is widely used in vegetation phenology extraction because of its simple calculation method. However, it is sensitive to noise, has poor robustness, and is not suitable for climate studies in different regions and vegetation types^45,46^. The change detection method extracts candidate parameters by directly detecting the change characteristics of the vegetation index time series curves^36^. In contrast, the change monitoring method is widely used and can effectively determine key parameters of vegetation phenology^47^. Partial vegetation, especially crops, undergoes two growing seasons due to natural environmental factors such as climate and soil. However, existing research focuses more on single growing season vegetation, leaving a gap in the study of phenology in two-season vegetation. There remains significant uncertainty regarding the extraction of phenological parameters in dual-season vegetation and their response mechanisms to preseason climate factors. Meanwhile, the accuracy of the parameters needs to be improved due to the lack of ground data calibration^48,49^. Therefore, this study was calibrated with ground-based phenology data to consider two-seasonal vegetation phenology parameters and to extract phenology parameters for different vegetation types.

China's vast expanse spans multiple climatic zones and hosts a diverse array of vegetation types^50^, positioning it as an unrivaled focal point for vegetation phenology research. This study concentrates on the region north of 30°N in China, recognized for its heightened sensitivity to global climate change and suitability for investigating climate impacts on vegetation phenology^51^. The NDVI seasonal patterns in this region exhibit pronounced shifts, facilitating the identification of critical climatic periods affecting vegetation. Moreover, satellite-derived vegetation indices in this area are less influenced by solar zenith angles, ensuring enhanced accuracy in extracted vegetation phenology periods relative to southern regions^52^. Agriculture in this northern region predominantly follows one-season and two-season rotations due to simpler cultivation practices^32^. Thus, our study sheds light on the characteristics of mono- and bi-seasonal vegetation phenology and their responses to climate variables within China's northern region, beyond 30°N.

This study centers on China's middle and high latitudes, employing multi-source data encompassing GIMMS NDVI, relative humidity (RH), shortwave radiation (SR), maximum temperature (Tmax), and minimum temperature (Tmin) spanning from 1982 to 2014. Our aim is to elucidate the relationship between phenology and climate from a comprehensive, macroscopic perspective. Notably, we examine the phenology of two-seasonal vegetation, a facet often overlooked in prior research on vegetation phenology-climate relationships. Over the 1982–2014 timeframe, we hypothesized that easily ignored climate factors may have an effect on vegetation phenology. We analyze how the combined responses of Start of Season (SOS) and End of Season (EOS) relate to pre-season hydrothermal conditions. Furthermore, we explore the interplay between phenology and climatic factors across diverse vegetation types, including Deciduous Needle Leaf Forest (DNF), Deciduous Broadleaf Forest (DBF), Mixed Forest (MF), Grassland (GL), Savanna (SA), and Cropland (CL). This research holds paramount significance in identifying the determinants of vegetation phenology, comprehending the mechanisms steering phenological shifts, and gauging the repercussions of climate change on terrestrial ecosystems.

## Methods

### Study area

**Figure 1:**
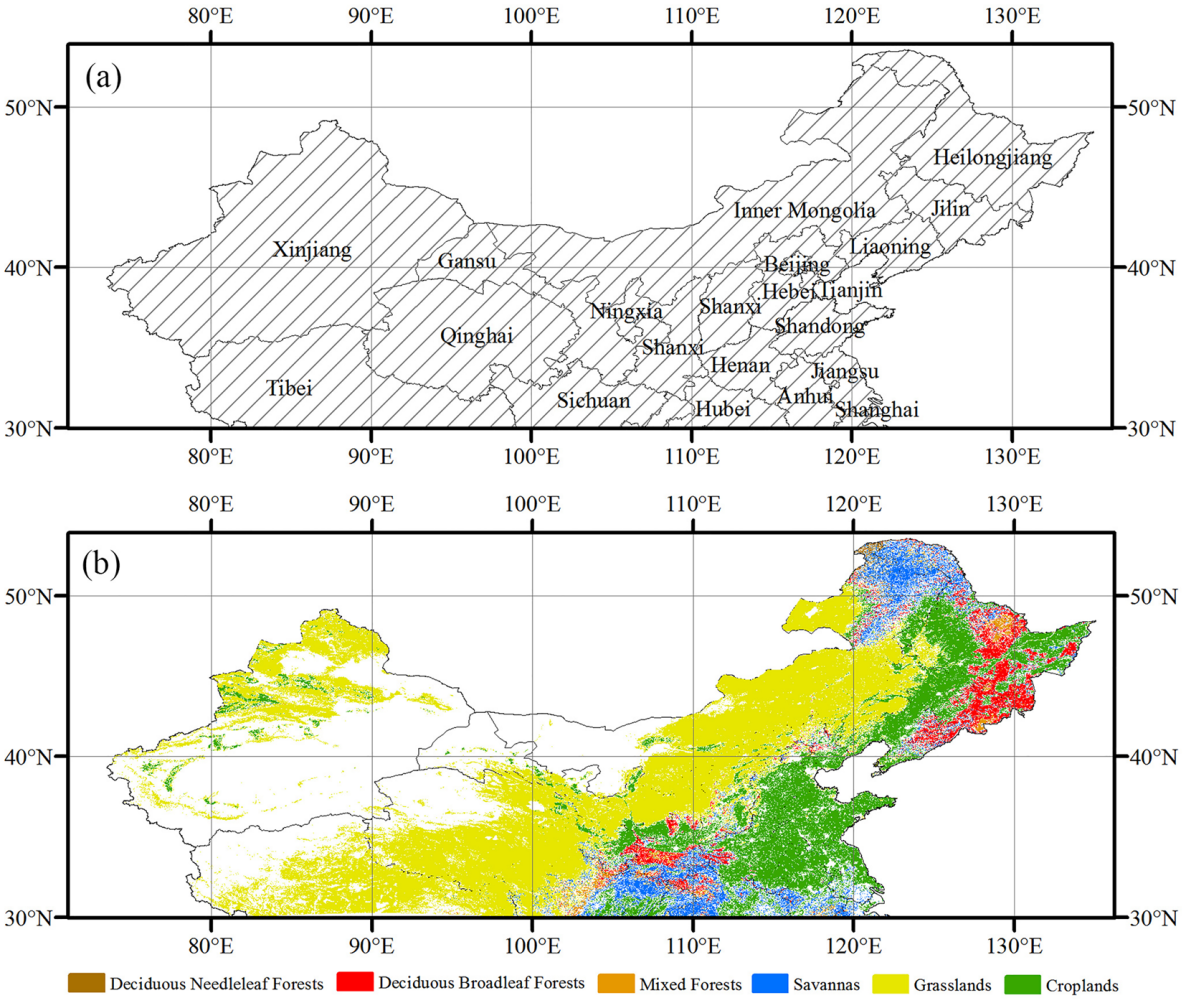
The northern part of China located above the latitude of 30°N (**a**). Spatial patterns of different vegetation types (**b**). The basemap was generated by cartopy package (version 0.21.1) in Python 3.9.12 (https://scitools.org.uk/cartopy/docs/latest/index.html).

The study area encompasses the temperate region of China situated north of the 30°N latitude, as illustrated in Fig. 1a. To mitigate the influence of changes in non-vegetated land use categories, this study relies on data obtained from the Land Cover Climate Modeling Grid product (MCD12C1) from MODIS satellites, which specifically excludes grids indicating land use changes between 2001 and 2014. Regions characterized as non-vegetated land, including barren land, water bodies, urban and built-up areas, permanent snow and ice, as well as permanent wetlands, were excluded from our analysis. Additionally, vegetation types consisting of fewer than 100 image elements were also excluded. The spatial distribution of vegetation types within the study area is shown in Fig. 1b.

### Datasets and preprocessing

We used the National Oceanic and Atmospheric Administration (NOAA) Global Inventory Monitoring and Modeling System (GIMMS) NDVI product, characterized by a spatial resolution of 0.0833333°, to compute key phenology parameters of vegetation spanning from 1982 to 2014. These data products, subject to rigorous quality control measures, have the advantage of offering long time series data and have gained widespread utilization within the field of vegetation phenology research^53–55^. The extracted vegetation phenology metrics, namely Start of Season (SOS) and End of Season (EOS), underwent validation for accuracy against ground phenology datasets^32^.

We selected easily neglected climate factors to explore their relationship with vegetation phenology. In particular, in the context of global diurnal asymmetric warming, Tmax and Tmin show different trends^56^, and despite the fact that there is a link between Tmax and Tmin in most cases, their effects on vegetation phenology have been found to be different^29–31,57^. To align the phenology parameters with the study area, raster data with a spatial resolution of 0.83333° were generated from maximum and minimum temperature, relative humidity, and downward shortwave radiation data. These meteorological data were sourced from 786 monitoring stations spanning the years 1982 to 2014 and were obtained through the China Meteorological Data Network (CMDN) (http://data.cma.cn/), employing spline interpolation techniques^58^.

The Land cover data were derived from the Land Cover Climate Modeling Grid product (MCD12C1) from MODIS satellites in 2001 and 2014. These data were resampled to a spatial resolution of 0.83333° using the nearest neighbor method. And non-vegetation land types and vegetation types with fewer than 100 pixels were excluded from the data to obtain the vegetation classification data we need.

### Calculation of phenological parameters

The NDVI time series for each pixel was reconstructed using the Savizky-Golay (SG) filter, preserving pixels with unimodal and bimodal structures (pixels with bimodal structures are candidates for two-season vegetation). The reconstructed NDVI curve is then differentiated to compute the second derivative, enabling the determination of SOS and EOS^59^. Simultaneously, based on ground phenological data, limited time windows for SOS and EOS are defined to accurately extract phenological parameters for different vegetation types. If the SOS and EOS of a particular pixel align with the time window of a single vegetation phenology, it is associated with that vegetation type. If the SOS and EOS of both growing seasons coincide with the time window of two-season vegetation from ground observations, it is classified as two-season vegetation^32^.

### Statistical analysis

The Pearson correlation analysis was conducted to explore the relationships between SOS and EOS and various climate factors at the pixel level, considering different preseason lengths. The preseason climate factor was defined as the average climate factor for the N days leading up to the phenology period within the growing season. In some studies, N is referred to as the "duration of the time window", representing the length of the preseason, and it typically ranges from 1 to 180^60^.

**Figure 2:**
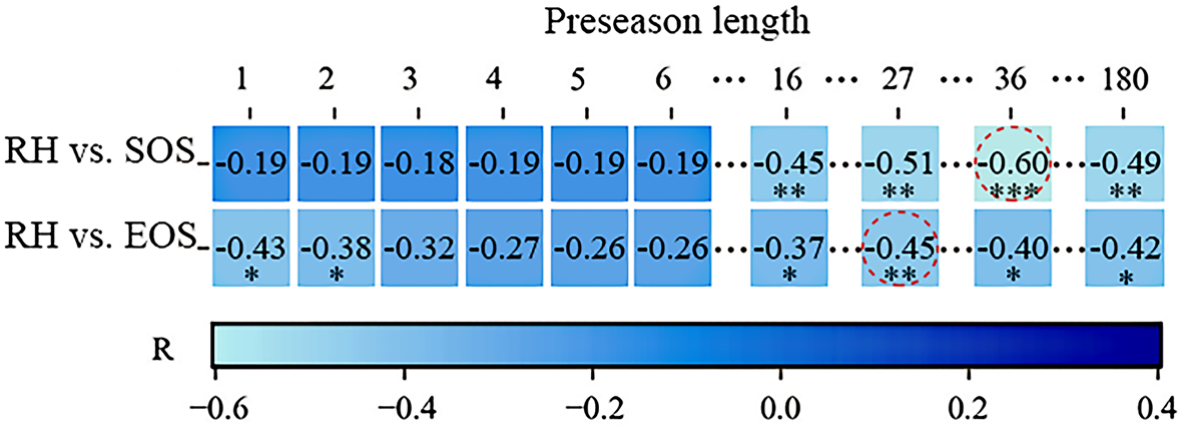
The matrix of correlation coefficients. The red dashed circle indicates the highest value within each row, with its associated preseason length representing the optimal length (significance levels are indicated by ***for *p* ≤ 0.001, **for *p* ≤ 0.01, and *for *p* ≤ 0.05).

As shown in Fig. 2, we selected the preseason length that exhibited the highest absolute Pearson's correlation coefficient as the optimal preseason length for both vegetation phenology and each preseason climate factors.

## Results

### Optimal preseason length characterization

**Figure 3:**
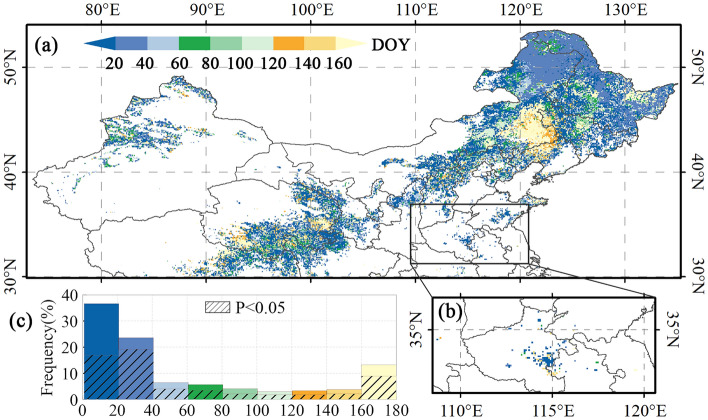
Spatial patterns of SOS and preseason Tmax optimal preseason length in (**a**) single-season vegetation, first season of two-season vegetation, and (**b**) second season of two-season vegetation. (**c**) Histograms of the optimal preseason length. The basemap was generated by cartopy package (version 0.21.1) in Python 3.9.12 (https://scitools.org.uk/cartopy/docs/latest/index.html).

**Figure 4:**
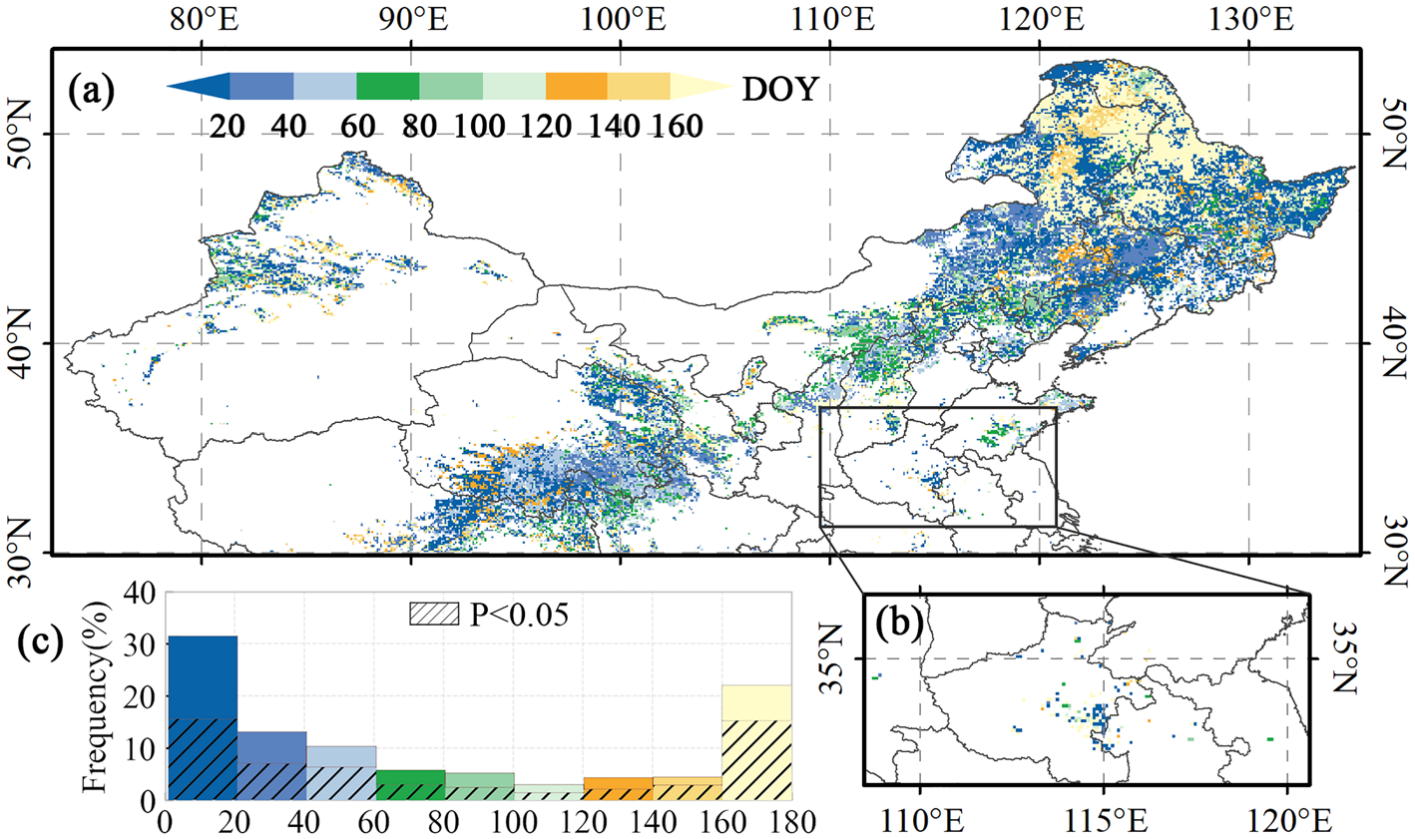
Spatial patterns of EOS and preseason Tmax optimal preseason length in (**a**) single-season vegetation, first season of two-season vegetation, and (**b**) second season of two-season vegetation. (**c**) Histograms of the optimal preseason length. The basemap was generated by cartopy package (version 0.21.1) in Python 3.9.12 (https://scitools.org.uk/cartopy/docs/latest/index.html).

The spatial pattern of the optimal preseason length between SOS, EOS and preseason Tmax is shown in Figs. 3 and 4. The preseason length in which Tmax presents the greatest impact on SOS is concentrated in the 0–60 days. The optimal preseason length in northern Inner Mongolia and northern Heilongjiang is dominated by 20–40 days. In contrast, the optimal preseason length in the intersection between the Liaoning, the Jilin, and the Inner Mongolia provinces is dominated by 120–180 days. The preseason length where the preseason Tmax exhibits the largest impact on EOS is centered on the 0–20 days and 160–180 days. The optimal preseason length is 140–180 days in large areas of northern Heilongjiang and northern Inner Mongolia.

### Characterization of vegetation phenology in response to climatic factors

**Figure 5:**
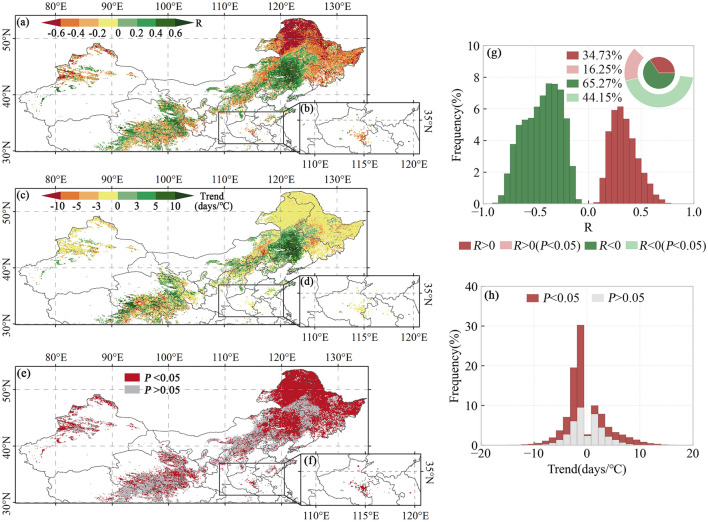
Spatial patterns of R-values (**a**, **b**), slopes (**c**, **d**), and P-values (**e**, **f**) of SOS vs. preseason Tmax for single-season vegetation (**a**, **c**, **e**), first season of two-season vegetation (**a**, **c**, **d**), and second season of two-season vegetation (**b**, **d**, **f**). Histograms of frequency distributions of R-values (**g**) and trends (**h**). The basemap was generated by cartopy package (version 0.21.1) in Python 3.9.12 (https://scitools.org.uk/cartopy/docs/latest/index.html).

The spatial distribution of vegetation SOS and preseason Tmax correlations, trend slopes, and significance are shown in Fig. 5. Vegetation SOS is significantly correlated with the preseason Tmax in 60.4% of the region of interest. Among them, about 16.25% of the areas show a significant positive correlation, and 44.15% have a significant negative correlation. Significant negative correlation dominates in northern Inner Mongolia, Heilongjiang and Jilin provinces. The SOS is generally significantly positively correlated with the preseason Tmax in areas such as the junction of Inner Mongolia and Liaoning. Significant negative correlations between SOS and preseason Tmax dominate in both the first and second seasons of the two-season vegetation.

**Figure 6:**
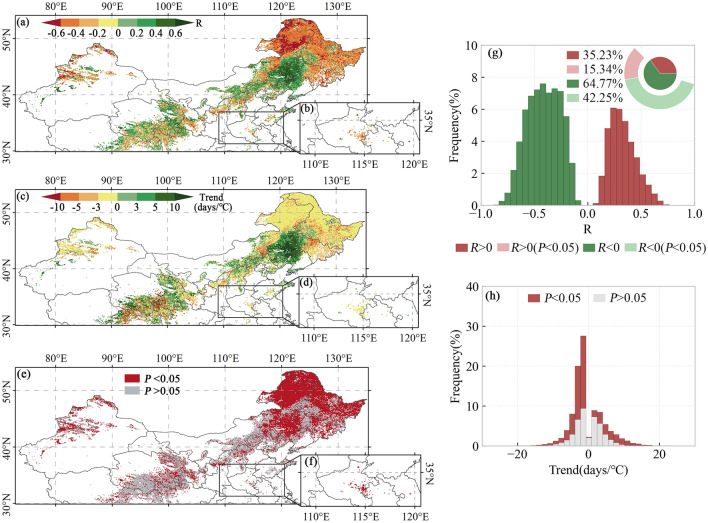
Spatial patterns of R-values (**a**, **b**), slopes (**c**, **d**), and P-values (**e**, **f**) of SOS vs. preseason Tmin for single-season vegetation (**a**, **c**, **e**), first season of two-season vegetation (**a**, **c**, **d**), and second season of two-season vegetation (**b**, **d**, f**)**. Histograms of frequency distributions of R-values (**g**) and trends (**h**). The basemap was generated by cartopy package (version 0.21.1) in Python 3.9.12 (https://scitools.org.uk/cartopy/docs/latest/index.html).

The spatial distribution of vegetation SOS and preseason Tmin correlations, trend slopes, and significance are shown in Fig. 6. The vegetation SOS is significantly correlated with preseason Tmin in 57.59% of the areas in the study area. Among them, about 15.34% of the areas show a significant positive correlation, and 42.25% have a significant negative correlation. Significant negative correlation dominates in northern Inner Mongolia, Heilongjiang and Jilin provinces. The SOS is generally significantly positively correlated with the preseason Tmin in areas such as the junction of Inner Mongolia and Liaoning. Negative correlations between SOS and preseason Tmin were dominant in both the first and second seasons of the two-season vegetation.

**Figure 7.**
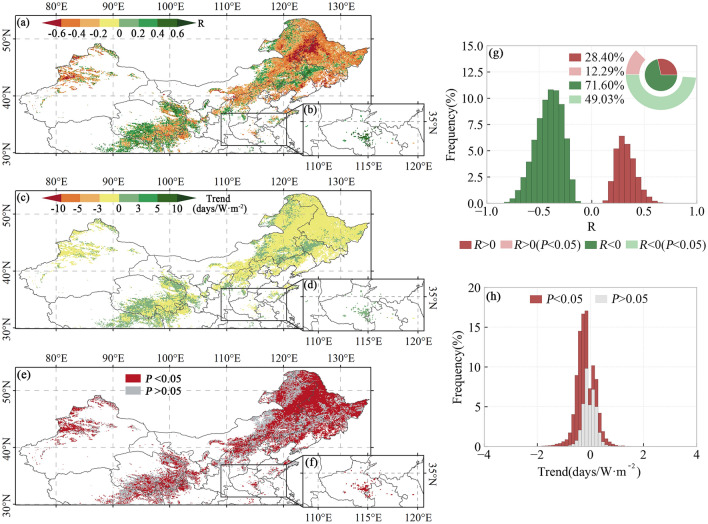
Spatial patterns of R-values (**a**, **b**), slopes (**c**, **d**), and P-values (**e**, **f**) of EOS vs. preseason SR for single-season vegetation (**a**, **c**, **e**), first season of two-season vegetation (**a**, **c**, **d**), and second season of two-season vegetation (**b**, **d**, **f**). Histograms of frequency distributions of R-values (**g**) and trends (**h**). The basemap was generated by cartopy package (version 0.21.1) in Python 3.9.12 (https://scitools.org.uk/cartopy/docs/latest/index.html).

The spatial distribution of vegetation EOS and preseason SR correlations, trend slopes, and significance is shown in Fig. 7. The vegetation EOS is significantly correlated with preseason SR in 61.32% of the studied regions. Among these, about 12.29% (49.03%) exhibit a significant positive (negative) correlation. The intersection between Inner Mongolia and Heilongjiang Province and other places is dominated by a significant negative correlation. Areas such as the intersection between Inner Mongolia and Liaoning and the southern part of the Qinghai Province generally present a significant positive correlation between EOS and preseason SR. Significant positive correlations are found between EOS and preseason SR in both the first and second seasons of the two-season vegetation.

### Response characteristics of different vegetation phenology to climatic factors

**Figure 8:**
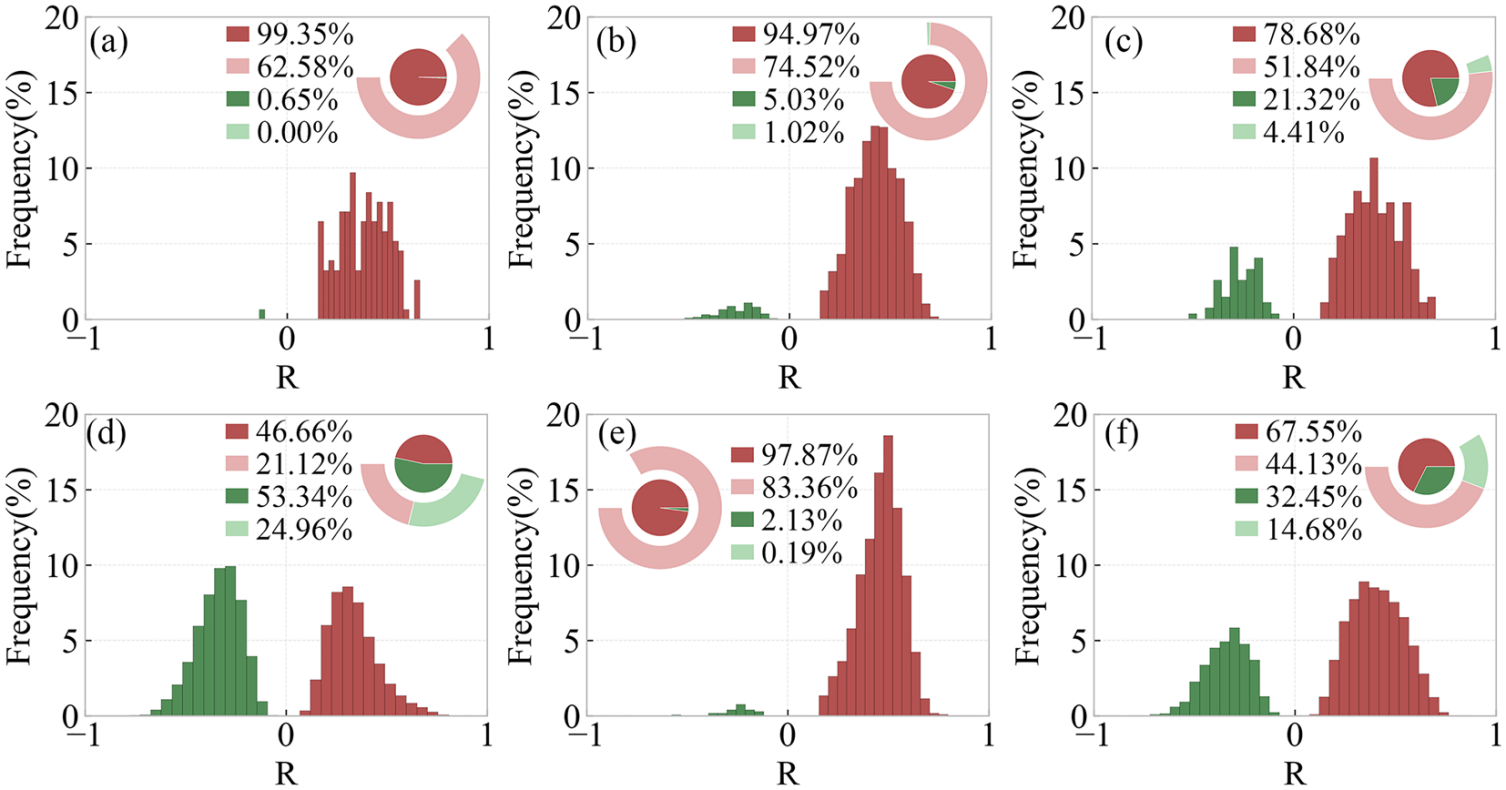
Frequency distribution of regions with different R-values of SOS and preseason RH for different types of vegetation including DNF (**a**), DBF (**b**), MF (**c**), GL (**d**), SA (**e**), CL (**f**).

Figure 8 shows the distribution of regions with different R-values of SOS and preseason RH for different types of vegetation. In DNF, DBF and SA, SOS and preseason RH mainly show a significant positive correlation, accounting for 62.58%, 74.52% and 83.36%, respectively. In MF and CL, SOS and preseason RH generally show a significant positive correlation, accounting for 51.84% and 44.13%, respectively, and some areas show a significant negative correlation, accounting for 4.41% and 14.68%, respectively. In GL, SOS is significantly positively correlated with pre-season RH in 21.12% of the areas and significantly negatively correlated with preseason RH in 24.96%.

**Figure 9:**
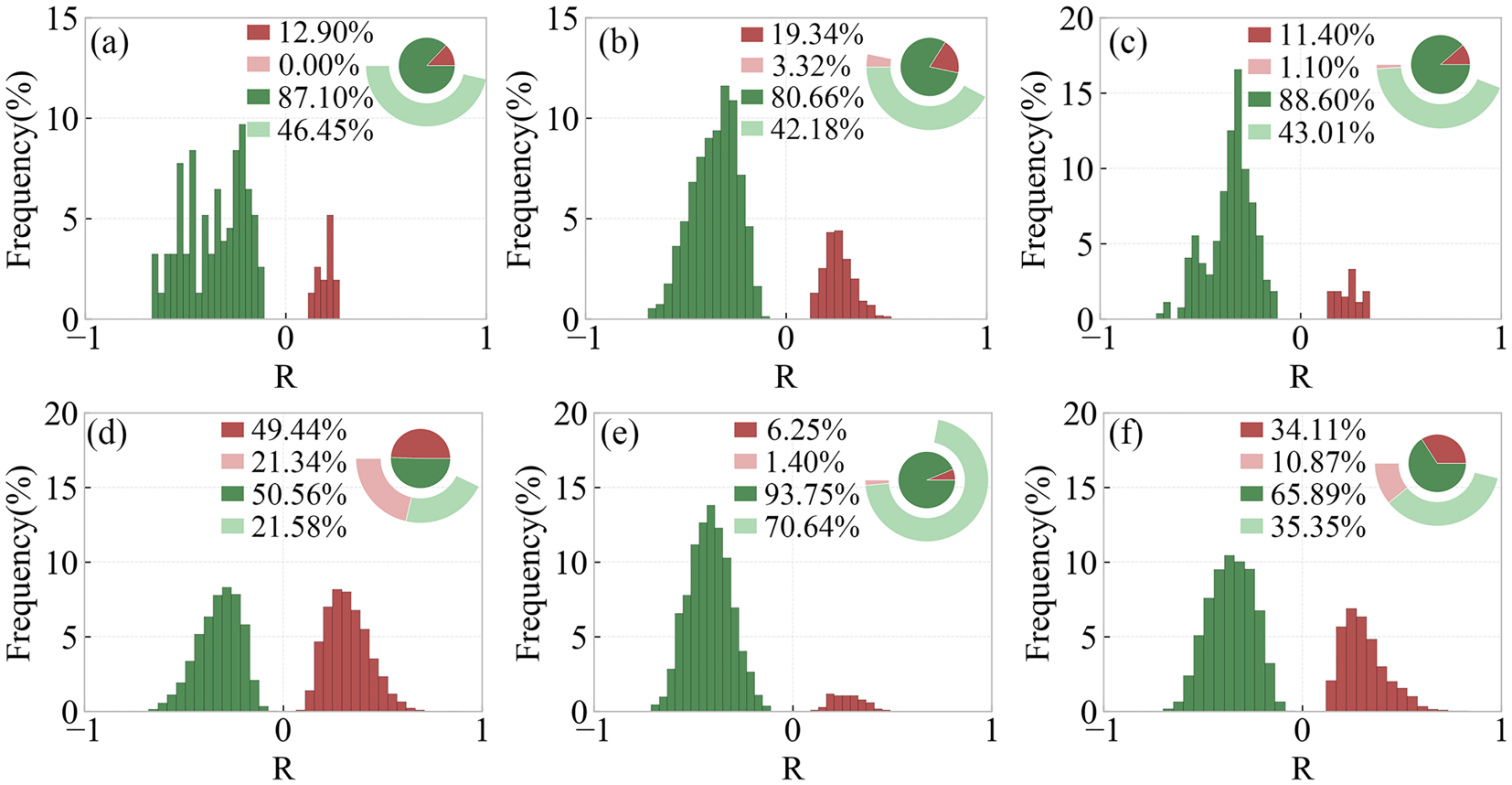
Frequency distribution of regions with different R-values of EOS and preseason RH for different types of vegetation including DNF (**a**), DBF (**b**), MF (**c**), GL (**d**), SA (**e**), CL (**f**).

Figure 9 shows the frequency distribution of regions with different R-values of EOS and preseason RH for different types of vegetation. In DNF, DBF, MF and SA, EOS shows a generally significant positive correlation with preseason RH, with percentages of 46.45%, 42.18%, 43.00% and 70.64%, respectively. In GL, 21.34% of the area shows a significant positive correlation between EOS and preseason RH, and 21.58% is significantly negative correlation between EOS and preseason RH. In CL, 10.87% of area EOS is significantly positively correlated with preseason RH, and 35.35% of area EOS is significantly negatively correlated with preseason RH.

**Figure 10:**
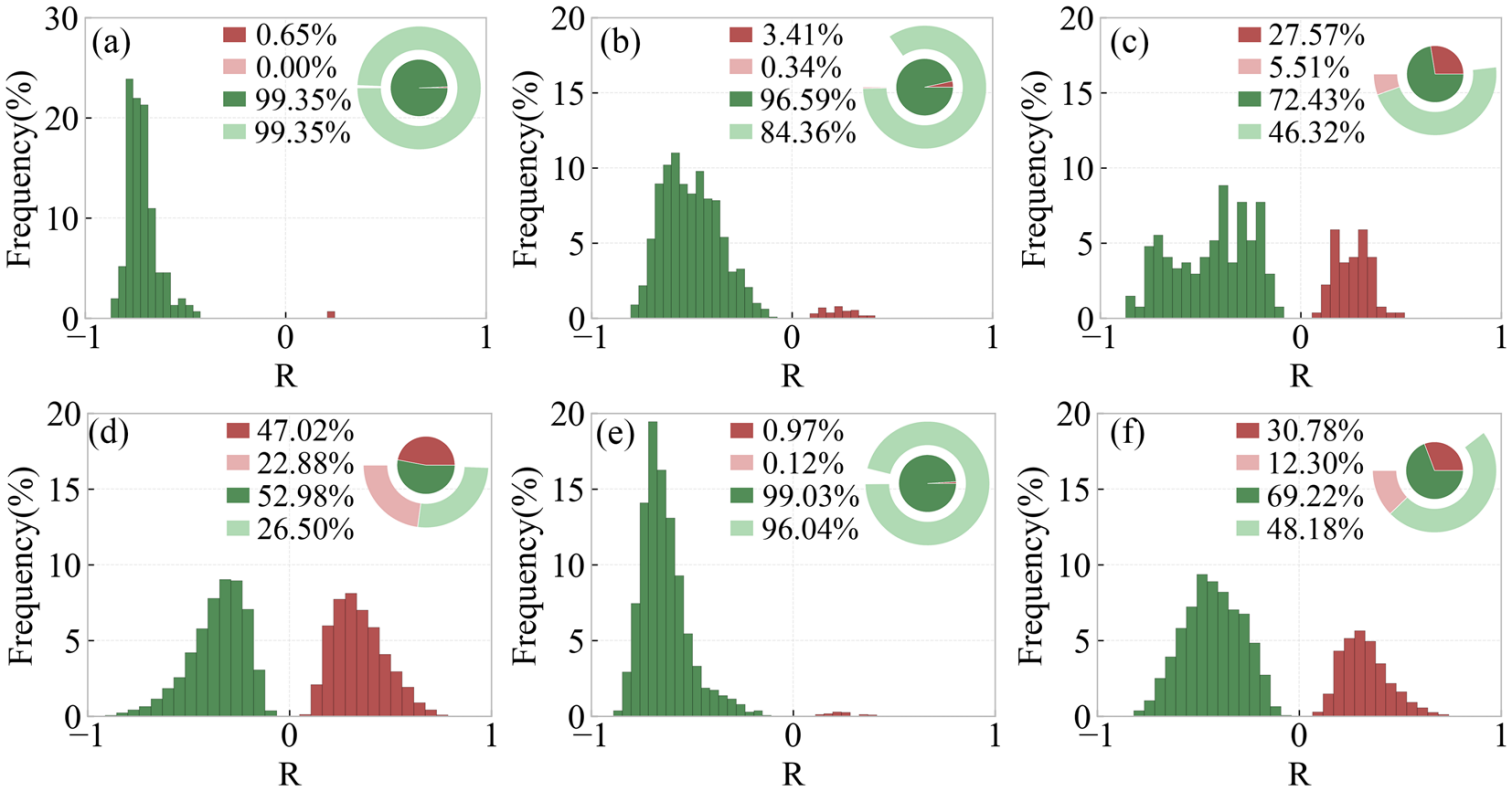
Frequency distribution of regions with different R-values of SOS and preseason Tmax for different types of vegetation including DNF (**a**), DBF (**b**), MF (**c**), GL (**d**), SA (**e**), CL (**f**).

Figure 10 shows the distribution of regions with different R-values of SOS and preseason Tmax for different types of vegetation. In DNF, DBF and savanna, SOS and preseason Tmax are significantly negatively correlated with each other, accounting for 99.35%, 84.36% and 96.04%, respectively. In MF and CL, SOS and preseason Tmax are significantly negatively correlated, with a larger percentage of 46.32% and 48.18%, respectively. In GL, EOS and preseason Tmax are significantly positively correlated in 22.88% of the area, and significantly negatively correlated in 26.5%.

**Figure 11:**
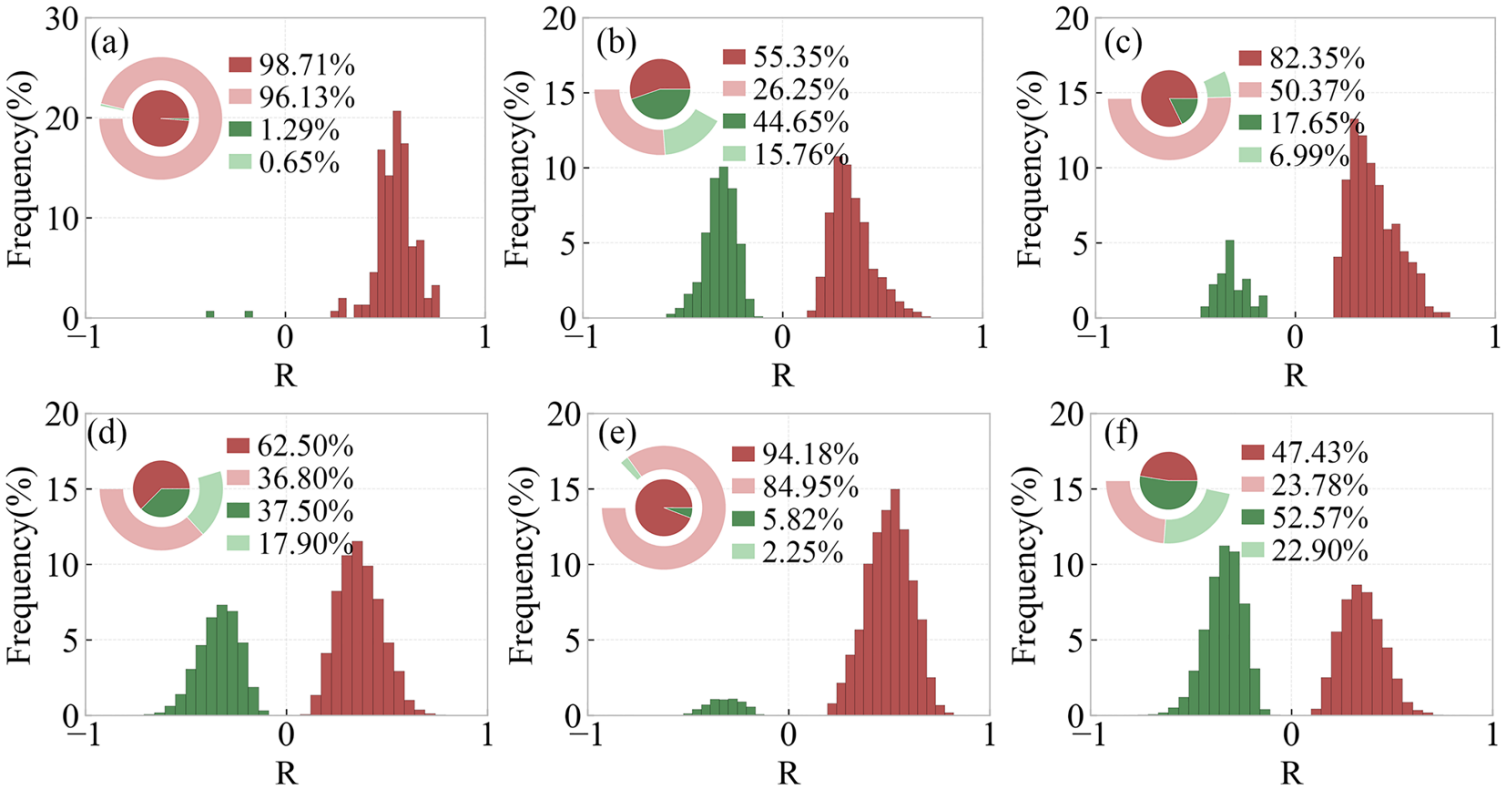
Frequency distribution of regions with different R-values of EOS and preseason Tmax for different types of vegetation including DNF (**a**), DBF (**b**), MF (**c**), GL (**d**), SA (**e**), CL (**f**).

Figure 11 shows the distribution of regions with different R-values of EOS and preseason Tmax for different types of vegetation. In DNF, SA and MF, EOS and preseason Tmax are significantly positively correlated, accounting for 96.13%, 84.95% and 50.37%, respectively. In DBF, EOS and preseason Tmax are significantly positively correlated in 26.25% of the areas and significantly negatively correlated in 15.76%. In GL, 36.8% of the area has a significant positive correlation between EOS and preseason Tmax, and 17.9% of the area has a significant negative correlation. In CL, 23.78% of the areas have a significant positive correlation between EOS and preseason Tmax and 22.9% of the areas have significant negative correlation.

**Figure 12:**
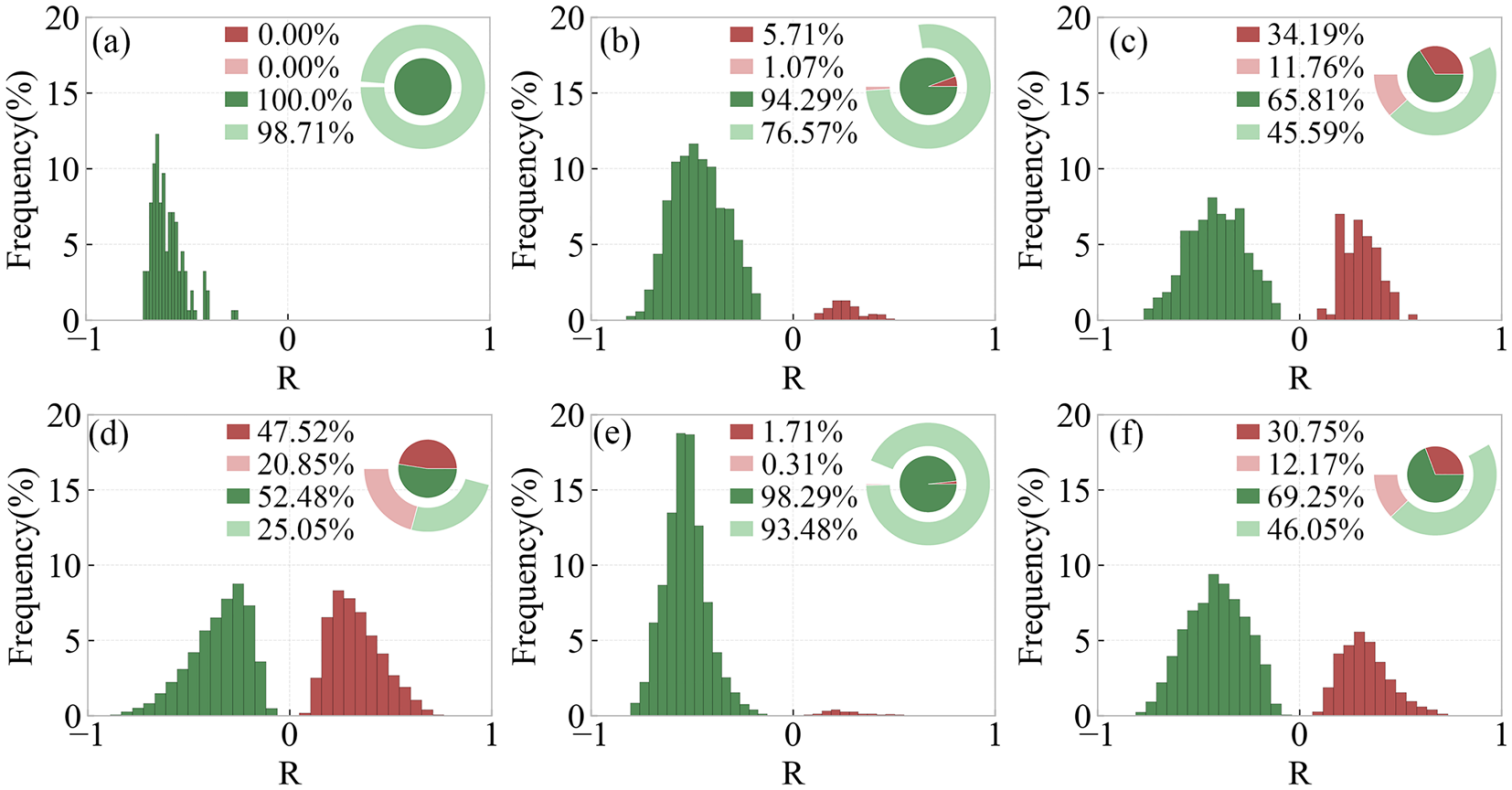
Frequency distribution of regions with different R-values of SOS and preseason Tmin for different types of vegetation including DNF (**a**), DBF (**b**), MF (**c**), GL (**d**), SA (**e**), CL (**f**).

Figure 12 shows the distribution of regions with different R-values of SOS and preseason Tmin for different types of vegetation. In DNF, DBF, SA, SOS and preseason Tmin are significantly negatively correlated with each other, accounting for 98.71%, 76.57% and 93.48%, respectively. In MF and CL, SOS and preseason Tmin are significantly negatively correlated with a larger percentage of 45.59% and 46.05%, respectively. In GL, SOS and preseason Tmin are significantly positively correlated in 20.85% of the areas and significantly negatively correlated in 25.05%.

## Discussion

When comparing RH and optimal preseason length of vegetation SOS (Fig. S1), we observe a rapid response of vegetation to changes in humidity. This response was particularly evident in regions such as northern Inner Mongolia and northern Heilongjiang, where the optimal preseason length consistently ranged from 20 to 40 days. Remarkably, this contrasted with the situation at the intersection of Liaoning, Jilin, and Inner Mongolia provinces, where the optimal preseason length was prolonged, ranging from 120 to 180 days. Similar results were observed when studying the effect of RH on EOS (Fig. S2). These differences may be attributed to the complex interactions of regional climate change. When considering the effects of SR, different trends emerge (Fig. S3). A preseason length of 0–20 days is critical for SOS effects, while a broader preseason length of 0–60 days affects EOS (Fig. S4). This difference may be attributed to differences in the sensitivity of SOS and EOS to SR. Additionally, the optimal preseason length for both SOS and EOS predominantly falls within 120–180 days, during the first and second seasons of the two-season vegetation. Biseasonal vegetation needs a longer period for photosynthesis and growth.It has been noted that SR predominates in the phenology of biseasonalvegetation^61^. This suggests that prolonged exposure to short-wave radiation is critical for the growth phase of the two-season vegetation. Solar radiation plays a key role in the photosynthesis and vegetation activity during the growing season^27^, for instance, its influence on plant growth response along with pre-season radiation intensity constitutes a significant factor affecting the spatial differentiation of phenology in temperate deciduous forests^62^.The preseason length in which both Tmax and Tmin have the greatest effect on SOS is primarily centered on days 0 to 60 (Fig. S5).

The onset of vegetation growth requires sufficient heat accumulation^63^. Generally, the temperature of the region needs to reach 0–5 °C to break the vegetation dormancy and start growth^64^. Therefore, increased temperature accelerates heat accumulation, promoting vegetation germination. The preseason of 0–60 days corresponds to the dormant period of vegetation, which is the main reason why the optimal preseason length for SOS and temperature is concentrated within this range. However, there are regions where the optimal preseason length for EOS and temperature is concentrated at 120–180 days. This may be due to the fact that the heat required for vegetation growth can accumulate rapidly due to warmer spring temperatures, leading to rapid vegetation growth. The climate effect on spring phenology further carries over to fall phenology through carryover effects^63,65^.

The relationship between vegetation SOS and EOS and preseason RH (Figs. S7, S8), SR, and temperature showed different trends across growing seasons and regions. RH influences seed germination and seedling growth in plants. Higher RH can facilitate rapid seed water absorption and germination under appropriate moisture conditions, favoring earlier crop growth^2^. However, excessive moisture may lead to overly wet soil, affecting seed oxygen supply root system growth, thereby delaying the onset of growth.

In some areas, we observed a significant positive correlation between preseason SR and SOS (Fig. S9), where an increase in SR may lead to a delay in the start date of vegetation growth. This could be attributed to increased SR resulting in higher surface temperatures, which may inhibit seed germination or seedling growth. Conversely, in other regions, we observed a significant negative correlation between preseason SR and SOS. Higher SR provides more energy to induce photosynthesis and plant growth. In most areas, there was a significant negative correlation between preseason SR and EOS (Fig. 7)^1,57^, indicating that an increase in SR accelerates EOS.This may be attributed to the fact that plants complete their life cycle more rapidly under increased light conditions^19^. When leaf absorption of light energy exceeds its photosynthetic capacity, it leads to an increase in plant leaf xanthophylls and anthocyanins, promoting leaf aging^66^. Simultaneously, the enhanced photosynthesis resulting from increased solar radiation accelerates carbon saturation and speeds up the aging process^67,68^. Additionally, preseason Tmax and Tmin were both significantly negatively correlated with vegetation SOS and significantly positively correlated with vegetation EOS in most areas (Figs. S10, S11)^1,19^. This implies that warmer temperatures in most areas will accelerate vegetation SOS and delay vegetation EOS^3^. Sufficient heat accumulation is required for the onset of vegetation growth, and warmer temperatures expedite this process, promoting vegetation germination^60,69^. Higher temperatures in summer and fall have been reported to enhance the activity of photosynthetic enzymes and reduce the rate of chlorophyll degradation, leading to a delay in EOS^54,70,71^. Furthermore, nighttime warming may contribute to the positive effects of preseason temperatures on EOS by reducing the risk of freezing due to low temperatures and slowing the leaf coloration process^72^.

This study reveals the diversity and complexity of vegetation responses to climate change by analyzing the relationship between phenology and easily ignored climate factors in various vegetation types. Both SOS and EOS were significantly positively correlated with RH in SA north of 30°N in China, indicating that RH was the dominant climatic factor for SA phenology. Significant negative correlations between SOS and Tmax and Tmin in DNF, DBF, and GL, suggesting that temperature strongly influences SOS in these three vegetation types. Additionally, the significant positive correlation between EOS and Tmax in DNF indicates that EOS in DNF is predominantly influenced by Tmin. How different vegetation types react to climatic factors is diverse, involving factors such as vegetation growth characteristics, ecological adaptations, and physiological mechanisms^29^. In various vegetation types, the link between phenology and climatic factors may be jointly influenced by several factors, such as growth strategy, photosynthetic efficiency, and water use efficiency^6,8,73^. The same vegetation type exhibited different patterns of relationships under different climate factors, suggesting a diversity of strategies for vegetation to adapt to climate change.

## Conclusion

In this research, we focused on the mid-to-high latitude region of China as our study area and we leveraged the GIMMS NDVI 3g long time series remote sensing dataset to extract climate parameters for different vegetation types at the raster level. Our analysis spanned the years 1982 to 2014, and our primary goal was to investigate the interplay between vegetation phenology and easily ignored climate elements. To accomplish this, we constructed correlation coefficient matrices between preseason RH, SR, Tmax, Tmin and vegetation phenology (SOS/EOS) at various raster locations. This allowed us to explore how different climate factors influence various vegetation phenology parameters within their optimal preseason length. The key findings of this study are as follows:

1. We observed that the optimal preseason lengths for RH, SR, Tmax, and Tmin, which exert the most significant influence on vegetation phenology, exhibited variability across different regions and growing seasons of vegetation. In most regions, the optimal preseason length influencing vegetation phenology fell within the range of 0–60 days.
2. Our analysis revealed diverse trends in the relationships between SOS, EOS, and preseason RH, SR, and temperature across different growing seasons and regions. Specifically, preseason RH exhibited a significant positive correlation with vegetation SOS in 37.86% of the areas, while it showed a significant negative correlation with vegetation EOS in 32.73% of the areas. Preseason SR was significantly negatively correlated with vegetation EOS in 49.03% of the areas. Furthermore, both preseason Tmax and Tmin displayed significant negative correlations with vegetation SOS in 44.15% and 42.25% of the areas, respectively.
3. The interactions between phenology and climate factors in various vegetation types were characterized by their diversity and complexity. Within the northern region of China, RH emerged as the dominant climatic factor influencing the phenology of SA vegetation. Temperature, on the other hand, exerted strong control over the SOS in three distinct vegetation types, namely DNF, DBF, and SA. Additionally, the EOS of DNF vegetation was predominantly governed by Tmax.

Overall, our research sheds light on the intricate relationships between climate and vegetation phenology, providing valuable insights into the dynamics of ecosystems in the mid-to-high latitude region of China.

### Supplementary Information

Supplementary Information.

## Data Availability

The datasets analyzed during the current study available from the corresponding author on reasonable request.

## Code availability

The source codes for the analysis of this study are available from the corresponding author upon reasonable request.
