# Supplementary material for: Understanding vegetation phenology responses to easily ignored climate factors in china's mid-high latitudes
Source: Sci Rep. 2024 Apr 16;14:8773. doi: 10.1038/s41598-024-59336-5 (PMC11021431; doi:10.1038/s41598-024-59336-5)
Supplement: Supplementary file 1 — Supplementary Information. [file 41598_2024_59336_MOESM1_ESM.pdf]

# Supplementary Information

*Understanding Vegetation Phenology Responses to Neglected Climate Factors in China's Mid-High Latitudes*

Qianfeng Wang[a, c]*, Huixia Chen[a], Feng Xu[a], Virgílio A. Bento[b], Rongrong Zhang[a], Xiaoping Wu[a], Pengcheng Guo[d, e]*

[a.] *Fujian Provincial Key Laboratory of Remote Sensing of Soil Erosion and Disaster Protection/College of Environmental & Safety Engineering, Fuzhou University, Fuzhou, 350116, China*

[b.] *Instituto Dom Luiz, Faculdade de Ciências, Universidade de Lisboa, 1749-016 Lisboa, Portugal*

[c.] *Key Lab of Spatial Data Mining & Information Sharing, Ministry of Education of China, Fuzhou, 350116, China*

[d.] *School of Ecology and Environment, Hainan University, Haikou, 570228, China*

[e.] *Hainan Guowei Eco Environmental Co., Ltd., Haikou, 570203, China;*

*The first corresponding author: Qianfeng Wang. E-mail: wangqianfeng@fzu.edu.cn

*The second corresponding author: Pengcheng Guo. E-mail: guopengcheng08@mails.ucas.ac.cn

## 1. Optimal preseason length characterization

The spatial pattern of the optimal preseason length between SOS and preseason RH is shown in Fig. S1. The preseason length where preseason RH has the greatest effect on SOS is more concentrated in the 0-40 days. The optimal preseason Length in northern Inner Mongolia and northern Heilongjiang is dominated by 20-40 days. In contrast, the optimal preseason length at the intersection of Liaoning, Jilin and Inner Mongolia provinces is dominated by 120-180 days. The preseason length of both the first and second seasons of the two-season vegetation, where preseason RH has the greatest effect on SOS, is 0-20 days. The spatial pattern of the optimal preseason length between EOS and preseason RH is shown in Fig. S2. The preseason length in which preseason RH has the greatest effect on EOS is days 0-20 and 160-180. The optimal preseason length at the intersection of Jilin, Heilongjiang and Inner Mongolia provinces is dominated by 160-180 days. The preseason length of both the first and second seasons of the two-season vegetation, where preseason RH has the greatest effect on EOS, is 0-20 days.

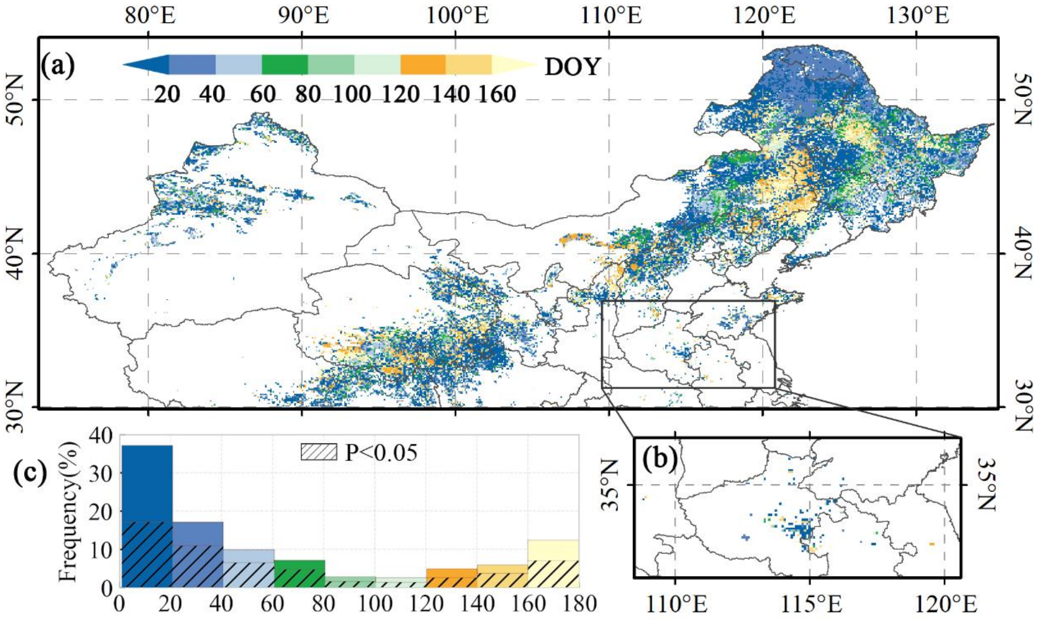


**Fig. S1.** Spatial patterns of SOS and preseason RH optimal preseason length in (a) single-season vegetation, first season of two-season vegetation, and (b) second season of two-season vegetation. (c) Histograms of the optimal preseason length. The basemap

was generated by cartopy package (version 0.21.1) in Python 3.9.12 (https://scitools.org.uk/cartopy/docs/latest/index.html).

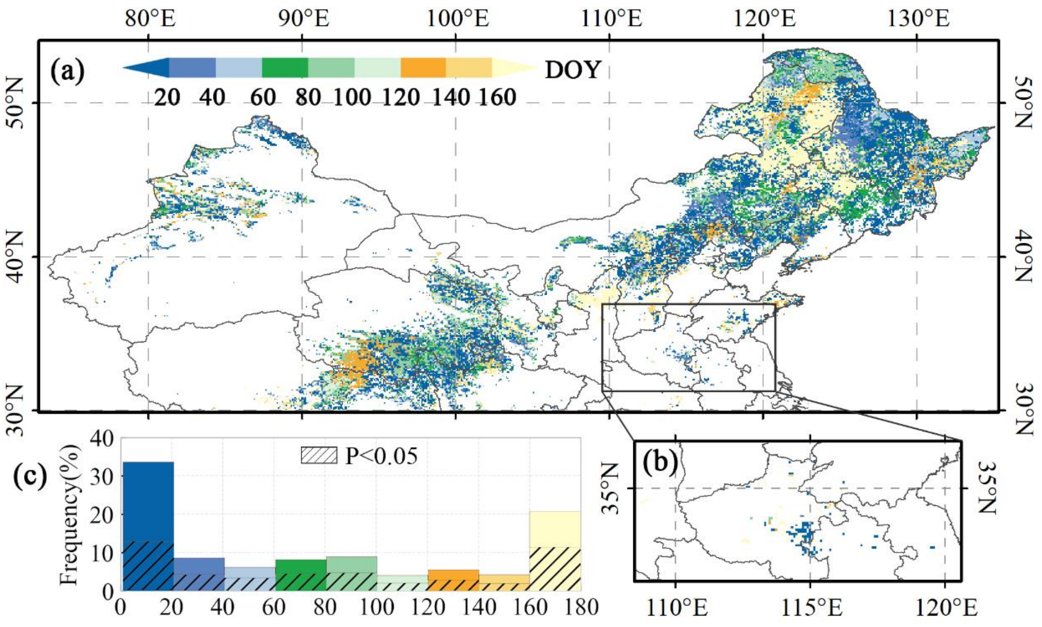


**Fig. S2.** Spatial patterns of EOS and preseason RH optimal preseason length in (a) single-season vegetation, first season of two-season vegetation, and (b) second season of two-season vegetation. (c) Histograms of the optimal preseason length. The basemap was generated by cartopy package (version 0.21.1) in Python 3.9.12 (https://scitools.org.uk/cartopy/docs/latest/index.html).

The spatial pattern of the optimal preseason length between SOS and preseason SR is shown in Fig. S3. The preseason length where preseason SR has the greatest effect on SOS is concentrated in the 0-20 days. The optimal preseason length is dominated by 20-40 days in the first season of the two-season vegetation, while 0-20 and 160-180 days dominate in the second season. The spatial pattern of the optimal preseason length between EOS and preseason SR is shown in Fig. S4. The preseason length where preseason SR has the greatest impact on EOS is more concentrated in the 0-60 days. The best preseason lengths in both the first and second seasons of the two-season vegetation were predominantly concentrated in the 120-180 days.

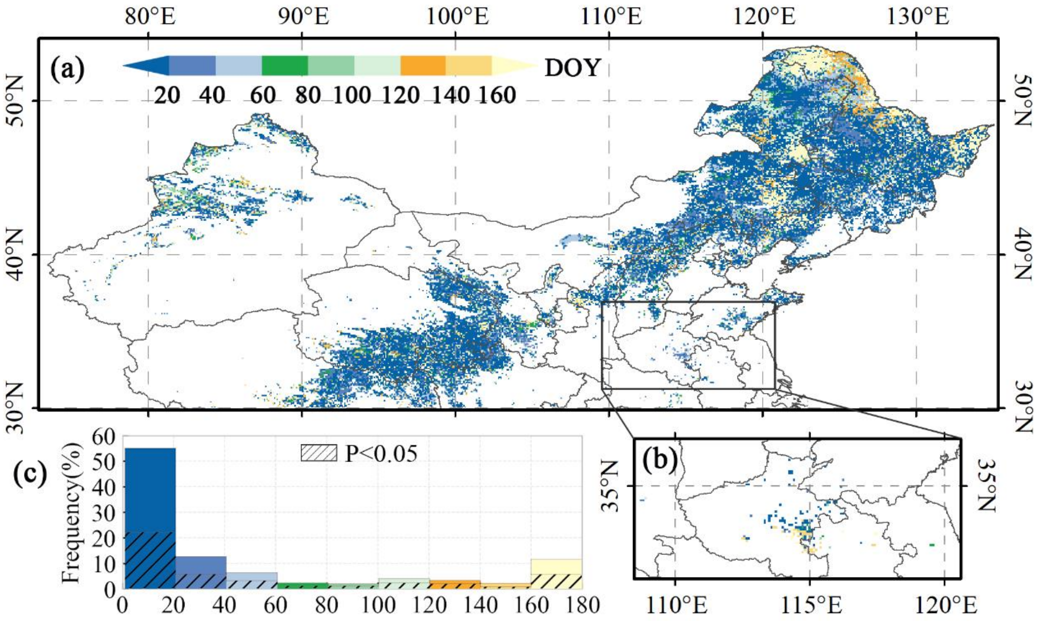


**Fig. S3.** Spatial patterns of SOS and preseason SR optimal preseason length in (a) single-season vegetation, first season of two-season vegetation, and (b) second season of two-season vegetation. (c) Histograms of the optimal preseason length. The basemap was generated by cartopy package (version 0.21.1) in Python 3.9.12 (https://scitools.org.uk/cartopy/docs/latest/index.html).

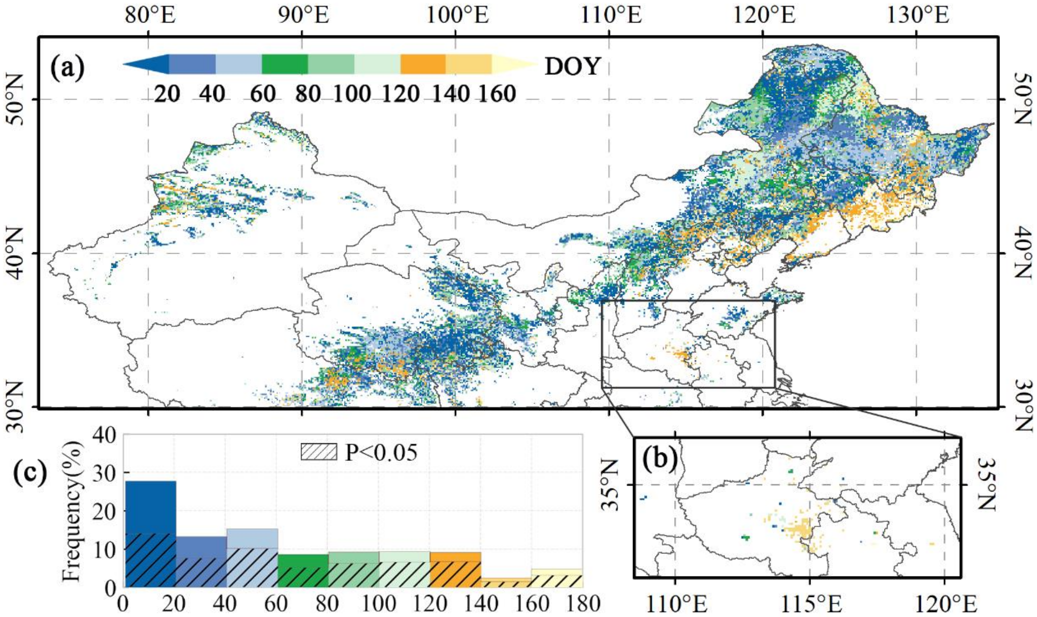


**Fig. S4.** Spatial patterns of EOS and preseason SR optimal preseason length in (a) single-season vegetation, first season of two-season vegetation, and (b) second season

of two-season vegetation. (c) Histograms of the optimal preseason length. The basemap was generated by cartopy package (version 0.21.1) in Python 3.9.12 (https://scitools.org.uk/cartopy/docs/latest/index.html).

The spatial pattern of the optimal preseason length between SOS, EOS and preseason Tmin is shown in Fig. S5, Fig. S6. The preseason length where preseason Tmin have the greatest effect on SOS is centred on days 0-60. The preseason length of 60-80 days, where preseason Tmin has the greatest impact on EOS, is more widely distributed than other climate factors.

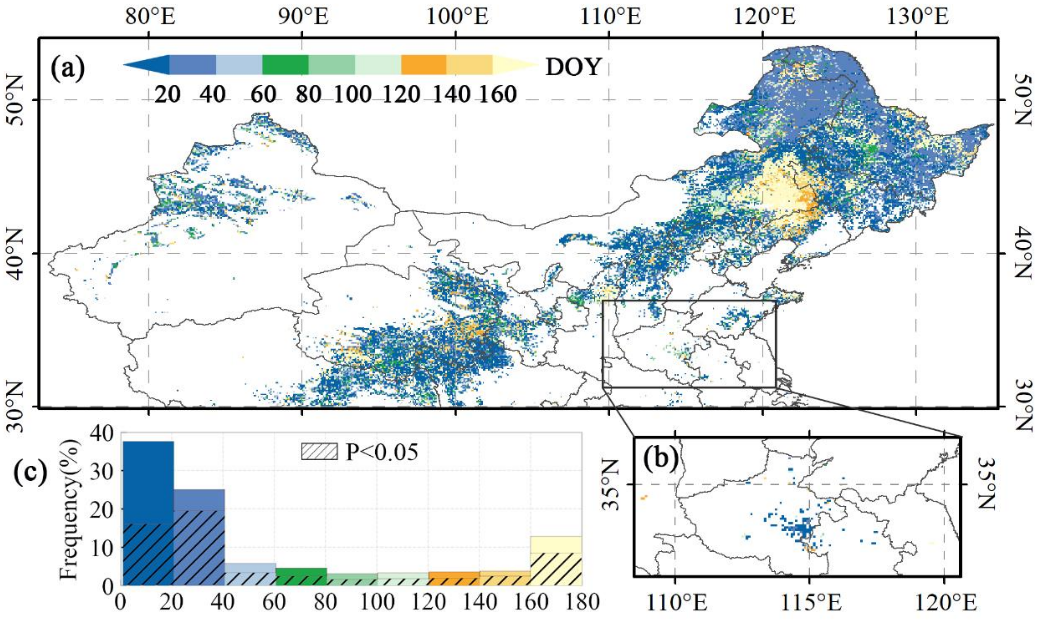


**Fig. S5.** Spatial patterns of SOS and preseason Tmin optimal preseason length in (a) single-season vegetation, first season of two-season vegetation, and (b) second season of two-season vegetation. (c) Histograms of the optimal preseason length. The basemap was generated by cartopy package (version 0.21.1) in Python 3.9.12 (https://scitools.org.uk/cartopy/docs/latest/index.html).

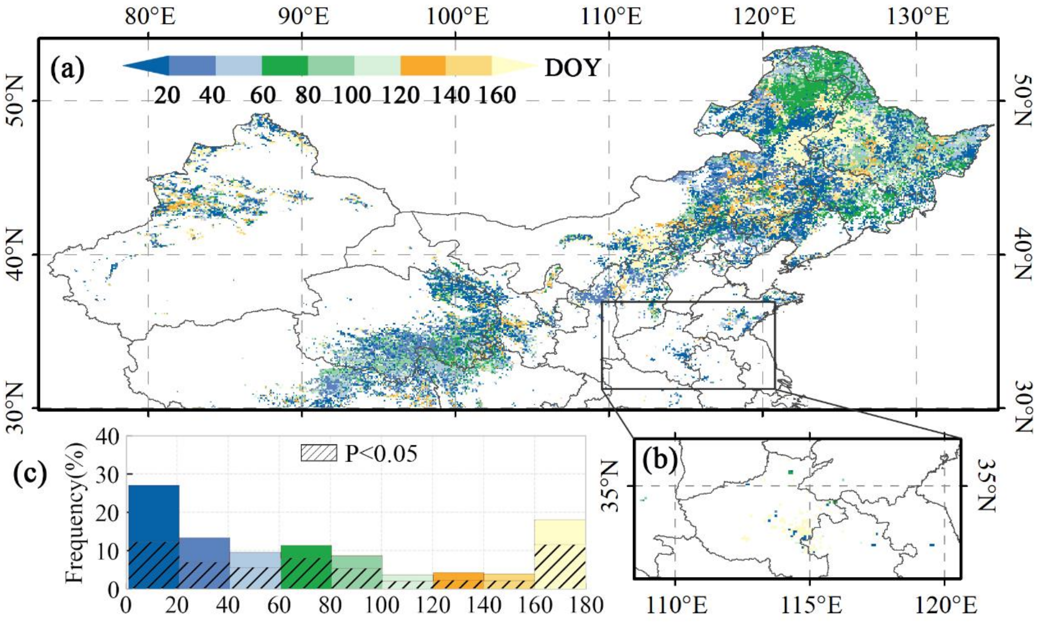


**Fig. S6.** Spatial patterns of EOS and preseason Tmin optimal preseason length in (a) single-season vegetation, first season of two-season vegetation, and (b) second season of two-season vegetation. (c) Histograms of the optimal preseason length. The basemap was generated by cartopy package (version 0.21.1) in Python 3.9.12 (https://scitools.org.uk/cartopy/docs/latest/index.html).

## 2. Characterization of vegetation phenology in response to climatic factors

The spatial distribution of vegetation SOS and preseason RH correlations, trend slopes, and significance are shown in Fig. S7. The vegetation SOS is significantly correlated with preseason RH in 55.81% of the areas in the study area. Out of these, about 37.86% of areas show a significant positive correlation, and 17.95% of areas have a significant negative correlation. Significant positive correlations dominate northern Inner Mongolia, Heilongjiang Province and Jilin Province. The SOS is generally significantly negatively correlated with preseason RH in the north part of Inner Mongolia and Liaoning Province. In areas such as the junction of Inner Mongolia and Liaoning and eastern Qinghai Province, SOS and preseason RH are generally significantly negatively correlated. Significant positive correlations between SOS and preseason RH dominate both the first and second seasons of the two-season vegetation.

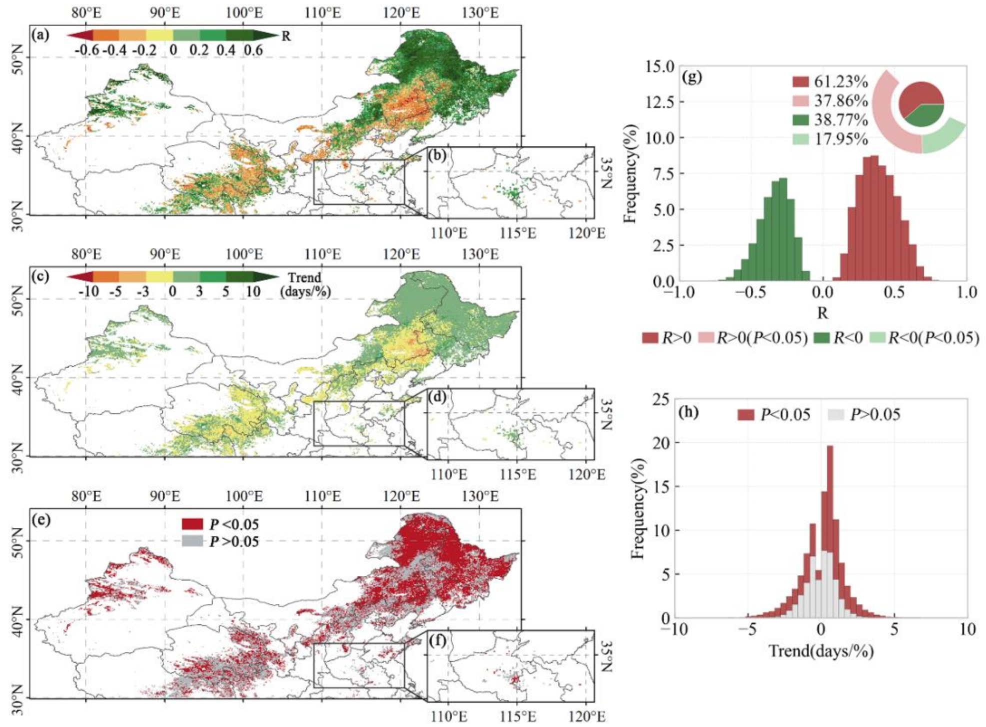


**Fig. S7.** Spatial patterns of R-values (a, b), slopes (c, d), and P-values (e, f) of SOS vs. preseason RH for single-season vegetation (a, c, e), first season of two-season vegetation (a, c, d), and second season of two-season vegetation (b, d, f). histograms of frequency distributions of R-values (g) and trends (h). The basemap was generated by cartopy package (version 0.21.1) in Python 3.9.12 (https://scitools.org.uk/cartopy/docs/latest/index.html).

The spatial distribution of vegetation EOS and preseason RH correlations, trend slopes, and significance are shown in Fig. S8. The vegetation EOS is significantly correlated with preseason RH in 48.07% of the areas in the study area. Among them, about 15.34% of the areas show a significant positive correlation, and 32.73% have a significant negative correlation. The northern part of Inner Mongolia and the eastern part of Heilongjiang Province is dominated by significant negative correlation. The EOS is generally significantly positively correlated with preseason RH in areas such as the junction of Inner Mongolia and Liaoning and the eastern part of Qinghai Province. Significant positive correlations between EOS and preseason RH are dominated in both the first and second seasons of bi-seasonal vegetation. In places such as eastern

Heilongjiang Province, preseason RH has a significant controlling effect on SOS but not on EOS.

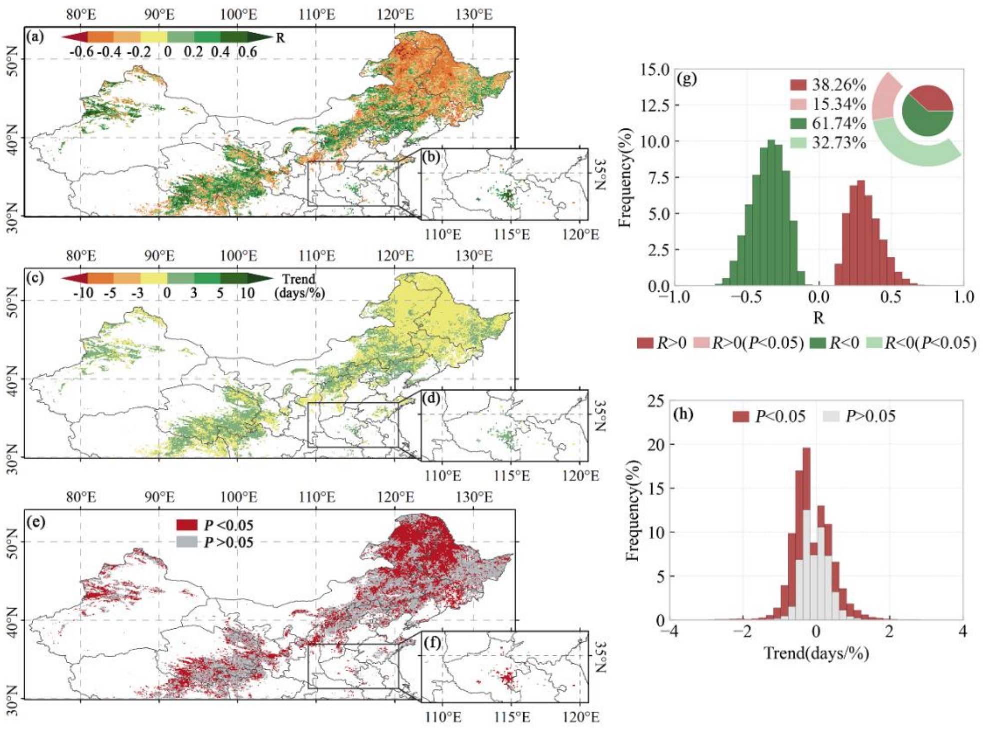


**Fig. S8.** Spatial patterns of R-values (a, b), slopes (c, d), and P-values (e, f) of EOS vs. preseason RH for single-season vegetation (a, c, e), first season of two-season vegetation (a, c, d), and second season of two-season vegetation (b, d, f). histograms of frequency distributions of R-values (g) and trends (h). The basemap was generated by cartopy package (version 0.21.1) in Python 3.9.12 (https://scitools.org.uk/cartopy/docs/latest/index.html).

The spatial distribution of vegetation SOS and preseason SR correlations, trend slopes, and significance are shown in Fig. S9. The vegetation SOS is significantly correlated with preseason SR in 44.61% of the areas in the study area. Among them, about 19.09% of the areas show a significant positive correlation, and 25.52% have a significant negative correlation. The northern part of Inner Mongolia and the north part of Heilongjiang Province is dominated by a significant negative correlation. The areas where Inner Mongolia meets Liaoning and the eastern part of Qinghai Province are generally negatively correlated between SOS and preseason SR. Significant negative

correlations between SOS and preseason SR were dominant in the first and second seasons of the two-season vegetation.

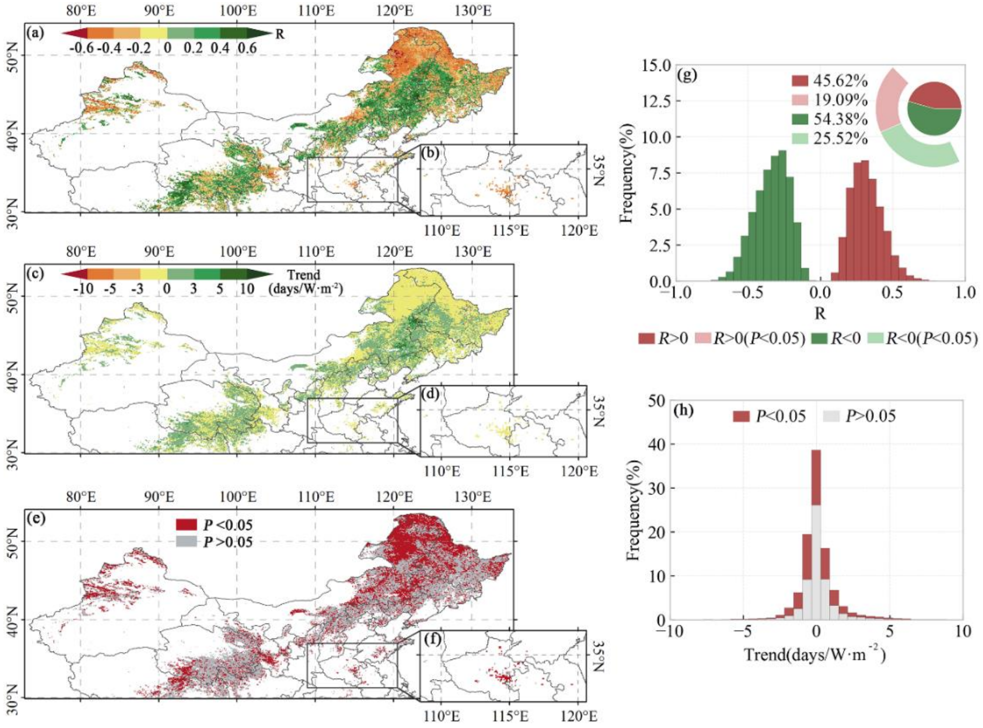


**Fig. S9.** Spatial patterns of R-values (a, b), slopes (c, d), and P-values (e, f) of SOS vs. preseason SR for single-season vegetation (a, c, e), first season of two-season vegetation (a, c, d), and second season of two-season vegetation (b, d, f). histograms of frequency distributions of R-values (g) and trends (h). The basemap was generated by cartopy package (version 0.21.1) in Python 3.9.12 (https://scitools.org.uk/cartopy/docs/latest/index.html).

The spatial distribution of vegetation EOS and preseason Tmax correlations, trend slopes, and significance are shown in Fig. S10. The vegetation EOS is significantly correlated with the preseason Tmax in 56.39% of the areas in the study area. Among them, about 38.57% of the areas show a significant positive correlation, and 17.82% have a significant negative correlation. Significant positive correlations are dominant in northern Inner Mongolia, western Heilongjiang Province, southern Qinghai Province, etc. The areas where EOS is generally significantly negatively correlated with the preseason Tmax are sporadically distributed. The negative correlation between EOS

and the preseason Tmax in both the first and second seasons of the two-season vegetation is dominated by the negative correlation, and the significance is not obvious.

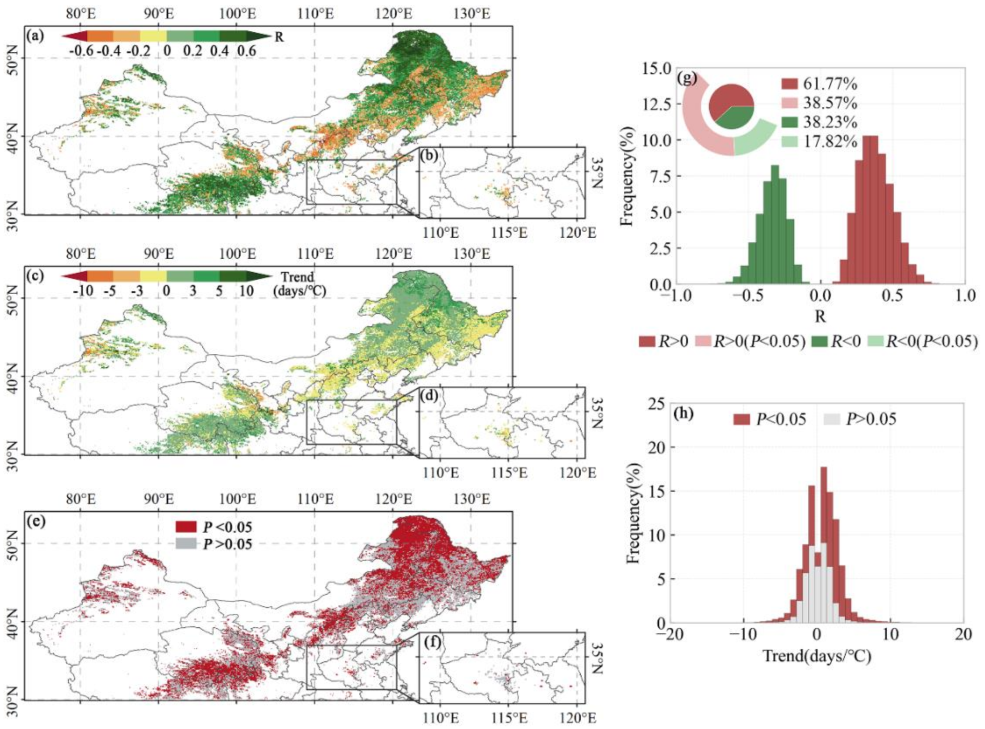


**Fig. S10.** Spatial patterns of R-values (a, b), slopes (c, d), and P-values (e, f) of EOS vs. preseason Tmax for single-season vegetation (a, c, e), first season of two-season vegetation (a, c, d), and second season of two-season vegetation (b, d, f). histograms of frequency distributions of R-values (g) and trends (h). The basemap was generated by cartopy package (version 0.21.1) in Python 3.9.12 (https://scitools.org.uk/cartopy/docs/latest/index.html).

The spatial distribution of vegetation EOS and preseason Tmin correlations, trend slopes, and significance are shown in Fig. S11. The vegetation EOS is significantly correlated with preseason Tmin in 56.37% of the areas in the study area. Among them, about 31.4% of the areas show a significant positive correlation, and 24.97% have a significant negative correlation. Significant negative correlation dominates in northern Inner Mongolia, Heilongjiang Province and Jilin Province. Where Inner Mongolia meets Heilongjiang, Jilin and Liaoning Provinces and in southern Qinghai Province,

EOS is generally significantly positively correlated with preseason Tmin. Significant negative correlations between EOS and preseason Tmin dominate in both the first and second seasons of the two-season vegetation.

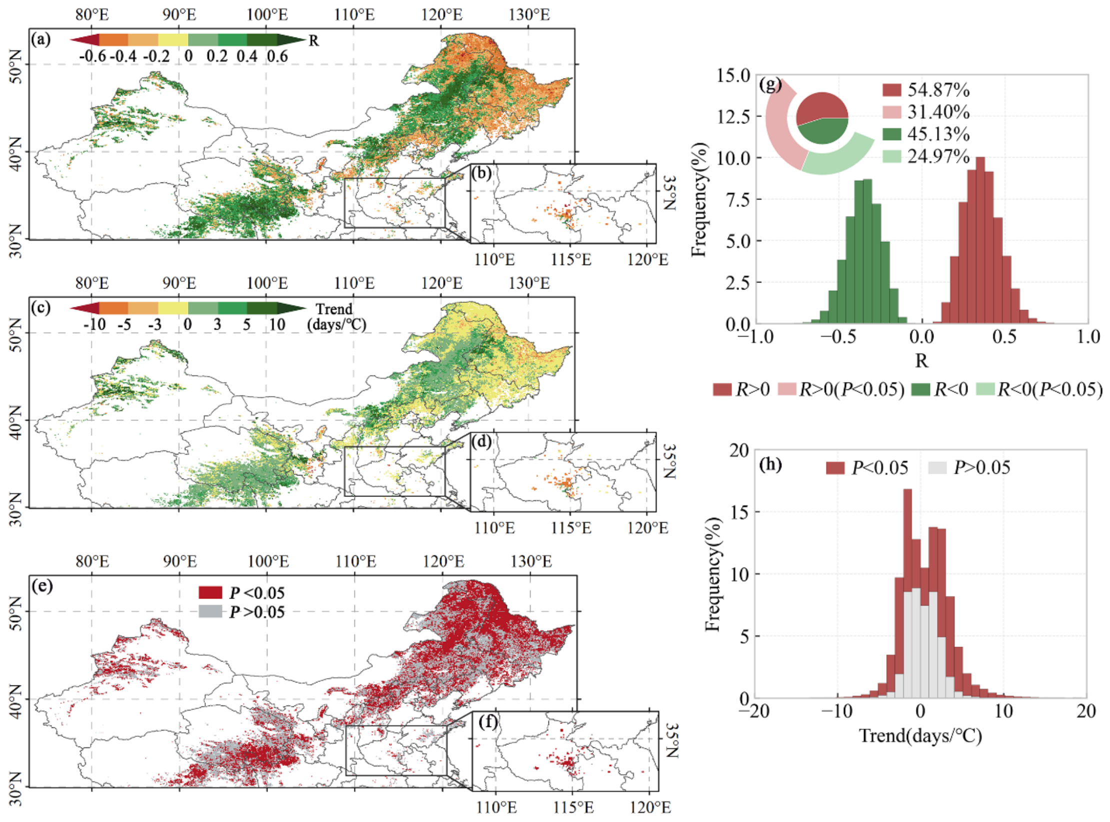


**Fig. S11.** Spatial patterns of R-values (a, b), slopes (c, d), and P-values (e, f) of EOS vs. preseason Tmin for single-season vegetation (a, c, e), first season of two-season vegetation (a, c, d), and second season of two-season vegetation (b, d, f). histograms of frequency distributions of R-values (g) and trends (h). The basemap was generated by cartopy package (version 0.21.1) in Python 3.9.12 (https://scitools.org.uk/cartopy/docs/latest/index.html).

## 3. Response characteristics of different vegetation phenology to climatic factors

Fig. S12 shows the frequency distribution of regions with different R-values of SOS and preseason SR for different types of vegetation. In DNF and GL, SOS and preseason SR are mainly significantly negatively correlated, accounting for 79.35% and 68.93%, respectively. In DBF, SOS and preseason SR are mainly significantly

negatively correlated in 33.57% of the areas. In MF, SOS and preseason SR are significantly negatively correlated in 36.03% of the areas and significantly positively correlated in 18.75% of the areas. In GL, SOS and preseason SR are significantly positively correlated in 24.49% of the areas and significantly negatively correlated in 14.16% of the areas. In CL, SOS and preseason SR are significantly negatively correlated in 24.31% of the areas and significantly positively correlated in 17.98% of the areas.

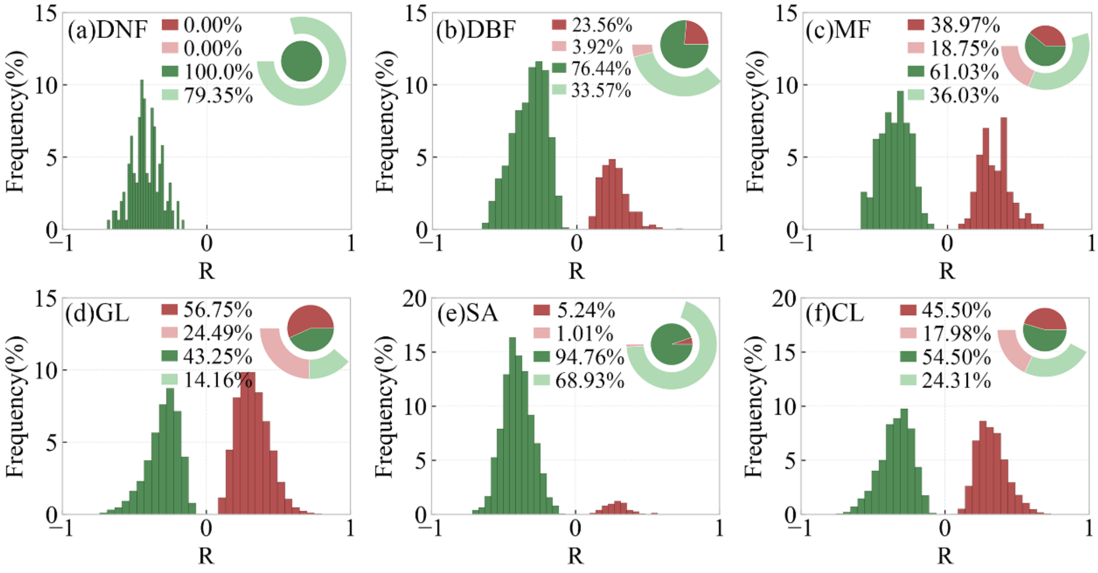


**Fig. S12.** Frequency distribution of regions with different R-values of SOS and preseason SR for different types of vegetation including DNF (a), DBF (b), MF (c), GL (d), SA (e), CL (f).

Fig. S13 shows the frequency distribution of regions with different R-values of EOS and preseason SR for different types of vegetation. In DBF, MF, SA and CL, EOS and preseason SR are mainly significantly negatively correlated, accounting for 73.84%, 56.25%, 57.49% and 55.72%, respectively. In DNF, EOS and preseason SR are significantly positively correlated in 20.65% of the areas and significantly negatively correlated in 13.55% of the areas. In GL, EOS and preseason SR are significantly negatively correlated in 38.57% of the areas and significantly positively correlated in 15.8% of the areas.

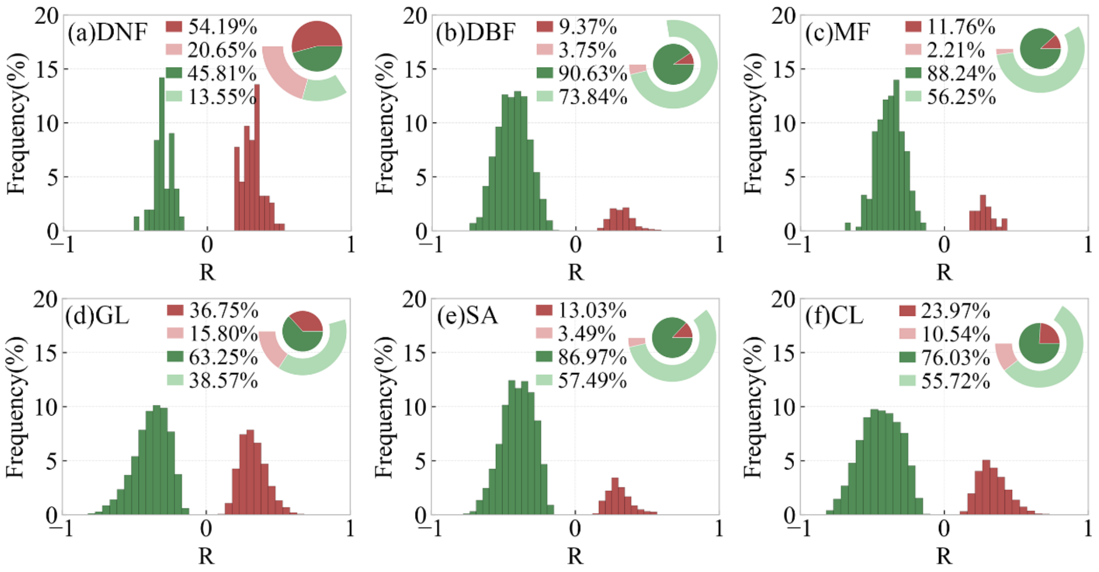


**Fig. S13.** Frequency distribution of regions with different R-values of EOS and preseason SR for different types of vegetation including DNF (a), DBF (b), MF (c), GL (d), SA (e), CL (f).

Fig. S14 shows the frequency distribution of regions with different R-values of EOS and preseason Tmin for different types of vegetation. In DBF, GL, MF and DNF, EOS and preseason Tmin are mainly significantly negatively correlated, accounting for 55.39%, 63.97%, 60.59% and 32.26%, respectively. In GL, EOS and preseason Tmin are mainly significantly positively correlated, accounting for 40.9%. In CL, EOS and preseason Tmin are significantly negatively correlated in 30.12% of the areas and significantly positively correlated in 22.75%.

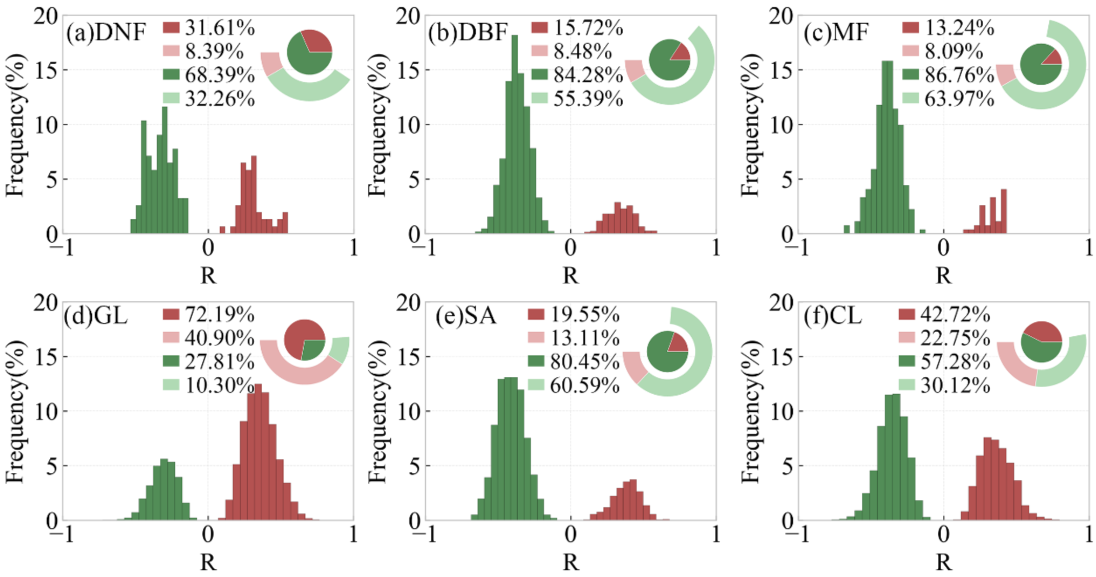


**Fig. S14.** Frequency distribution of regions with different R-values of EOS and preseason Tmin for different types of vegetation including DNF (a), DBF (b), MF (c), GL (d), SA (e), CL (f).
